# Interaction between N^6^-methyladenosine modification and the tumor microenvironment in colorectal cancer

**DOI:** 10.1186/s10020-023-00726-2

**Published:** 2023-09-22

**Authors:** Jiali Yao, Yeke Song, Xiaoping Yu, Zhijie Lin

**Affiliations:** 1 Department of Immunology, Institute of Translational Medicine, Medical College, Yangzhou University, Yangzhou, 225009 Jiangsu China; 2 Health Management Center, Affiliated Hospital of Yangzhou University, Yangzhou University, Yangzhou, 225009 Jiangsu China; 3 Jiangsu Key Laboratory of Experimental and Translational Non-Coding RNA Research, Yangzhou University, Yangzhou, 225001 China

**Keywords:** Colorectal cancer, m^6^A, Tumor microenvironment

## Abstract

The incidence and mortality of colorectal cancer (CRC) are rapidly increasing worldwide. Recently, there has been significant attention given to N^6^-methyladenosine (m^6^A), the most common mRNA modification, especially for its effects on CRC development. It is important to note that the progression of CRC would be greatly hindered without the tumor microenvironment (TME). The interaction between CRC cells and their surroundings can activate and influence complex signaling mechanisms of epigenetic changes to affect the survival of tumor cells with a malignant phenotype. Additionally, the TME is influenced by m^6^A regulatory factors, impacting the progression and prognosis of CRC. In this review, we describe the interactions and specific mechanisms between m^6^A modification and the metabolic, hypoxia, inflammatory, and immune microenvironments of CRC. Furthermore, we summarize the therapeutic role that m^6^A modification can play in the CRC microenvironment, and discuss the current status, limitations, and potential future directions in this field. This review aims to provide new insights into the molecular targets and theoretical foundations for the treatment of CRC.

## Introduction

Colorectal cancer (CRC) is a common malignancy of the digestive system, with its incidence and mortality increasing worldwide (Arnold et al. 2017). It ranks as the third deadliest tumor and the fourth most frequently diagnosed tumor in the world (Sung et al. 2021). Despite advancements in technology and treatment options, the estimated mortality of CRC patients remains high (Cao et al. 2021a). Various risk factors contribute to the development of CRC, including diet, intestinal metabolism, genetics, polyp lesions, and chronic inflammation. These factors are interconnected and cannot exist independently. For instance, a high-fat diet (HFD) not only stimulates the proliferation of intestinal mucosal and cancer cells but also inhibits the proliferation of lymphocytes in the lamina propria and weakens immune function. This leads to intestinal barrier dysfunction and dysregulation of intestinal metabolism (Yang et al. 2022; Ocvirk and O'Keefe 2021). Disturbances in intestinal metabolism further promote chronic inflammation and CRC through various enzymes and metabolites (Jackson and Theiss 2020).

The tumor microenvironment (TME) is a dynamic environment that plays a crucial role in tumor cell survival, growth, proliferation, and metastasis (Feingold et al. 1997). It consists of a complex and heterogeneous system comprising both cellular and non-cellular elements. The cellular components of the TME include cancer-associated fibroblasts (CAFs), T lymphocytes, B lymphocytes, NK cells, tumor-associated macrophages (TAMs), tumor-associated neutrophils (TANs), and endothelial cells (Arneth 2019). In addition, there are non-cellular components such as growth factors, cytokines, and extracellular matrix that surround the tumor cells. These components interact and form specific metabolic, hypoxia, inflammatory, and immune microenvironments in CRC. They are responsible for the regulation of various pro- and anti-tumor factors and play a role in the progression of the disease. The composition of TME is influenced by various signaling pathways and cytokine flow, which involve a variety of chemical modifications. These modifications include ubiquitination, 5-methylcytosine (m^5^C), N6-methyladenosine (m^6^A), and 7-methylguanosine (m^7^G) modifications. Among them, m^6^A modification has been the most extensively studied in recent years (Jordan et al. 2018), (Maybin et al. 2018), (Catana et al. 2015), (Wu and Dai 2017).

Recently, m^6^A modification has emerged as a new regulatory mechanism in eukaryotes and is one of the most common RNA methylation modifications. It is a reversible epigenetic modification that occurs in both mRNAs and non-coding RNAs (ncRNAs). The m^6^A modification is catalyzed by certain methyltransferases. Following the m^6^A modification, methylated binding proteins precisely identify and bind to the modified RNA. These modifications can be reversed, and demethylases are responsible for their removal (Han et al. 2023). By modulating these processes, m^6^A modifications have a profound impact on the fate and cellular functions of the modified RNA molecules, influencing RNA splicing, export, translation, and stability. Furthermore, they play a crucial role in almost all essential biological processes, including the malignant progression of tumors (Deng et al. 2018; Alarcon et al. 2015). The progression of tumors is highly dependent on the microenvironment in which they are located. Several recent studies have attempted to uncover the correlation between m^6^A and the TME (Han et al. 2019a; Wang et al. 2020a; Li et al. 2021a). The m^6^A methylation recognition protein YT521-B homology domain family protein 1 (YTHDF1) regulates the tumor immune microenvironment. Deletion of YTHDF1 enhances the anti-tumor activity of CD8^+^T cells and inhibits the translation efficiency of lysosomal histone proteases in dendritic cells (DCs) (Han et al. 2019a). Overexpression of the m^6^A methylesterase Methyltransferase-like 3 (METTL3) alters the metabolic microenvironment of gastric cancer and promotes malignant tumor progression and liver metastasis (Wang et al. 2020a). Furthermore, components of the tumor microenvironment have been found to regulate the expression of m^6^A methylation regulators. For example, the hypoxia-inducible factor-1α (HIF-1α) can regulate the abundance of m^6^A (Li et al. 2021a). These findings suggest that the tumor microenvironment also plays a role in the complex regulatory network of m^6^A modification. The interactions between m^6^A and the tumor microenvironment are critical for tumor progression.

Although the correlation between m^6^A modification and the TME has been extensively studied, there is a scarcity of insights into the variants, functional characteristics, TME associations, and related clinical implications of m^6^A regulators in CRC. This review aims to summarize and demonstrate the specific roles of m^6^A regulators in the metabolic, hypoxic, inflammatory, and immune microenvironments associated with CRC, based on their reciprocal regulation with the TME. Additionally, our review highlights the potential diagnostic and therapeutic value of m^6^A modifications in the TME, discusses current research gaps, and suggests novel directions for future investigations.

## Molecular composition of m^6^A

The molecular composition of m^6^A includes methyltransferase, demethylase, and recognition factor, also termed m^6^A “writers”, “erasers” and “readers” respectively. These proteins have the ability to add, delete, or recognize m^6^A modification sites, and impacting crucial biological processes. Any factor that influences the expression levels of “writers” and “erasers” will consequently affect the activity of m^6^A in cells, resulting in abnormal levels of m^6^A in tumors. On the other hand, “readers” play dominant role in post-modification regulation of target mRNAs (An and Duan 2022). The mechanisms by which m^6^A enzymes recognize and regulate mRNA levels of specific target proteins are as follows: (a) certain “readers” recruit eukaryotic initiation translation factors and bind m^6^A labeled mRNA to ribosomes,(b) specific transcripts are bound by certain “readers,” thereby affecting mRNA translation; (c) some “writers” directly interact with transcription factors to mediate mRNA cyclization; (d) through histone modification, some “writers” are recruited to specific mRNA regions (Liu et al. 2023). These m^6^A enzymes regulate the expression level of proto-oncogenes or tumor suppressor genes by influencing the transcription, maturation, translation and degradation of RNA, ultimately participating in the occurrence and development of tumors (He et al. 2019).

METTL3 was the first characteristic component of m^6^A "writers" to be identified (Bokar et al. 1997). Currently, it is believed to primarily function as an oncogene, promoting tumor progression by adding m^6^A modifications to key transcripts (Li et al. 2019). METTL14 is another active component of m^6^A "writers" that binds to METTL3, forming stable heterodimeric complexes (Liu et al. 2014). While METTL3 is the catalytically active subunit, METTL14 plays a crucial role in recognizing the substrate structure (Wang et al. 2016). The third active component of m^6^A “writers” is Wilms’ tumor 1-associated protein (WTAP), which lacks catalytic activity but is essential for the nuclear localization and interaction between METTL3 and METTL14 (Scholler et al. 2018). METTL16, a homolog of METTL3, regulates U6 small nuclear RNA (U6 snRNA) methylation (Ruszkowska 2021). As a protective gene, the expression level of METTL16 has been found to exhibit a positively correlated with overall survival of several cancers (Li et al. 2020a). Conversely, a distinct investigation revealed that elevated expression of METTL16 was associated with a poor survival rate in patient with breast cancer (Zhang et al. 2020). Additionally, other components of m^6^A “writers” include virlike m^6^A methyltransferase associated (VIRMA/KIAA1429), RNA-binding motif protein 15 (RBM15), Cbl proto-oncogene, E3 ubiquitin-protein ligase-like 1 (CBLL1) and zinc finger CCCH-Type containing 13 (ZC3H13) (Jiang et al. 2021). VIRMA/KIAA1429 has been implicated in the malignant proliferation of CRC cells (Li et al. 2023a). CBLL1 is involved in the development of inflammatory bowel disease, and its dysregulation is associated with the inflammatory microenvironment of CRC (Roca-Lema et al. 2022). ZC3H13 has been demonstrated to suppress CRC invasion and proliferation by deactivating the Ras-ERK signaling pathway (Zhu et al. 2019).

In the current study, the m^6^A “erasers” consist of three main proteins: fat mass and obesity-associated protein (FTO), α-ketoglutarate-dependent dioxygenase homolog 5 (ALKBH5), and ALKBH3 (Huang et al. 2021; Dai et al. 2018). Jia et al*.* were the first to identify the demethylation activity of FTO in vitro against abundant m^6^A residues in RNA, indicating the reversible nature of m^6^A modification and its dynamic regulation (Jia et al. 2011).

Due to the multiple tumors signaling pathways FTO interacts with, the expression and effects of FTO in various tumors, whether it promotes or suppresses tumor growth, remain a subject of controversy. ALKBH5 was proven to have demethylation activity for the first time in 2013 (Zheng et al. 2013). Both ALKBH5 and FTO belong to the α-ketoglutarate-dependent dioxygenase family and catalyze m^6^A demethylation in an α-ketoglutarate-dependent manner (Marcinkowski et al. 2020). ALKBH3, a recently discovered m^6^A demethylase, regulates tumor progression by inducing tRNA demethylation and the production of corresponding miRNAs and proteins (Chen et al. 2019).

**Fig. 1:**
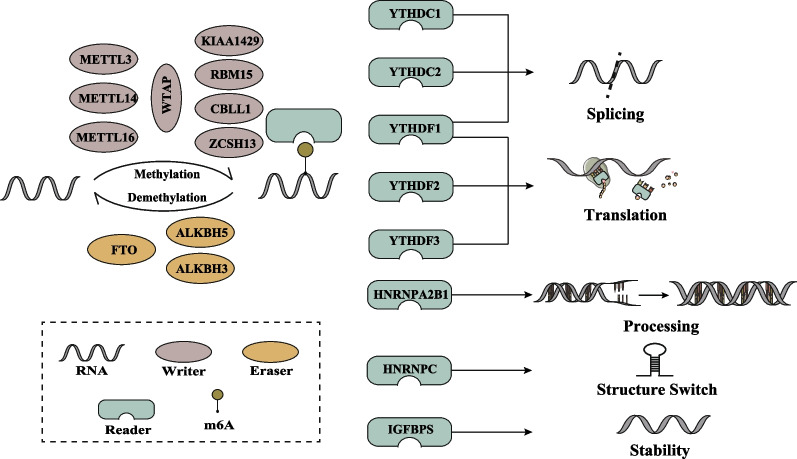
Classification and composition of m^6^A regulators. m^6^A “writers” include METTL3, METTL14, WTAP, METTL16, KIAA1429, RBM15, RBM15B, CBLL1, ZC3H13, which are responsible for structure stabilization and catalysis. m^6^A “erasers” include FTO, ALKBH5, ALKBH3, which are responsible for demethylation. HNRNPA2B1, HNRNPC, and IGF2BPs are responsible for recognizing m^6^A sites and mediating RNA splicing, translation, processing, structural transformation, and stabilization

The m^6^A “readers” activate downstream effector proteins or complexes in the trans conformation through recognition, which is an essential part of the biological function of m^6^A modifications (Yang et al. 2018; Allis and Jenuwein 2016). YT521-B homology domain family proteins 1–3 (YTHDF1-3) are three parallel sequences of the YTHDF family and they all have structural domains that selectively bind to m^6^A and are responsible for enhancing translation and degradation of mRNAs (Xiao et al. 2015; Li et al. 2017a). YTH domain-containing protein 1 (YTHDC1) promotes exon inclusion by recruiting the pri-mRNA splicing factor SRSF3 and blocking SRSF10 from binding to mRNA (Xiao et al. 2016). YTH domain-containing protein 2 (YTHDC2) can preferentially bind m^6^A-modified mRNA and affect its stability (Lma et al. 2020). In addition, several members of the heterogeneous nuclear ribonucleoprotein (HNRNP) family have also been shown to act as m^6^A "readers", such as HNRNPA2B1 and HNRNPC. They can regulate miRNA or mRNA abundance by processing m^6^A-modified RNA transcripts (Alarcón et al. 2015b; Lv et al. 2021). Moreover, insulin-like growth factor 2 mRNA-binding proteins (IGF2BPs) (including IGF2BP1/2/3), and the IGF-II mRNA-binding proteins (IMPs) family are also considered as m^6^A “readers” that can recognize specific m^6^A sequences targeted to mRNA for transcription (Huang et al. 2018). Figure 1 illustrates the classification and composition of m^6^A regulators.

## m^6^A modification and TME of CRC

### Metabolic microenvironment

The progression of CRC is closely related to the intestinal metabolic microenvironment, which includes factors such as intestinal flora, glucose metabolism, and lipid metabolism. It is believed that consuming high levels of dietary fiber and maintaining a stable intestinal metabolism can reduce the risk of CRC (Slavin 2008).

Recent study has revealed that the m^6^A methyltransferase METTL3 is responsible for regulating the cell cycle protein E1 (CCNE1) in CRC cells (Zhu et al. 2020). METTL3 promotes CRC proliferation by methylating the m^6^A site on CCNE1 mRNA. However, the intestinal microbial metabolite butyrate reduces m^6^A levels in CRC cells, reversing this process. Conversely, overexpression of METTL3 can counteract the inhibitory effects of butyrate on CRC progression (Zhu et al. 2020). In addition, the presence of *Enterotoxigenic Bacteroides fragilis* (ETBF) can downregulate miR-149-3p, leading to intestinal inflammation and promoting CRC. This process is dependent on METTL14-mediated m^6^A modification (Cao et al. 2021b). These finding suggest that there is a reciprocal relationship between the gut microbial metabolic environment and m^6^A regulators, which is closely linked to CRC progression. Another important factor in CRC is the protein KIAA1429, which targets HK2 mRNA to accelerate aerobic glycolysis and the production of malignant phenotypes in CRC (Li et al. 2022a). METTL3 also induces glycolysis and enhances CRC proliferation by promoting PTTG3P, which relies on IGF2BP2 for recognition its m^6^A binding site (Zheng et al. 2021). IMP2, a member of the m^6^A "readers", modifies the RNA metabolism of ZFAS1 through m^6^A modification. This process influences the energy metabolism of cell mitochondria, promotes CRC proliferation, and suppresses apoptosis of CRC cells (Lu et al. 2021). Concerning lipid metabolism, significant alterations have been observed in CRC patients. The enzyme DEGS2, which plays a crucial role in lipid metabolism, promotes CRC proliferation and metastasis in an m^6^A-dependent manner (Guo et al. 2021). In addition, chemoresistance is a major factor contributing to the failure of CRC chemotherapy. Drug-resistant cells undergo metabolic reprogramming to regulate the metabolic microenvironment within CRC. METTL3 has been shown to regulate glucose metabolism in CRC by enhancing the expression of LDHA and mediating resistance of CRC cells to 5-FU (Zhang et al. 2022a).

**Fig. 2:**
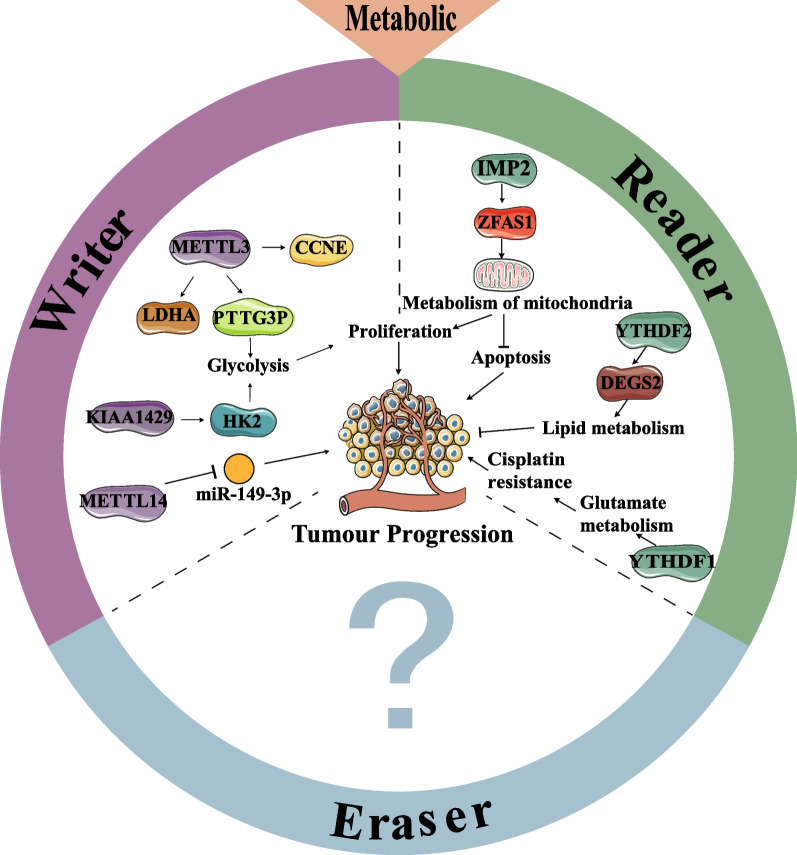
m^6^A modifications interact with the CRC metabolic microenvironment. m^6^A “writers” METTL3 promotes CRC proliferation by targeting LDHA and PTTG3P to regulate glycolysis. KIAA1429 promotes CRC proliferation by increasing the expression of HK2. METTL14 promotes CRC progression by inhibiting miR-149-3p. m^6^A “readers”, IPM2 targets ZFAS1 to mediate mitochondrial metabolism and inhibit CRC proliferation. YTHDF1 promotes CRC chemoresistance by mediating glutamate metabolism. YTHDF2 targets DEGS2 to regulate CRC lipid metabolism and inhibit CRC progression. The role of m^6^A “erasers” in the metabolic microenvironment in which CRC resides is still unclear

Together, m^6^A modifications play a bridging role that links the metabolic microenvironment to the progression of CRC. Certain components of the metabolic microenvironment in which CRC resides can influence its progression by affecting m^6^A modification. Current study specifically focuses on the interaction between intestinal microbe and m^6^A modification. Some of the intestinal flora implicated in CRC, such ads *Enterococcus faecalis* and *Helicobacter hepaticus*, are also responsible for producing numerous metabolites. However, whether these components are directly related to m^6^A modification requires further investigation. In the case of the glycolipid metabolic environment of CRC, m^6^A modifications only regulate this process in one direction. It is still unknown whether the metabolic pattern and the metabolites themselves also affect m^6^A modification in CRC. Figure 2 illustrates the role of m^6^A regulators in the metabolic microenvironment of CRC.

### Hypoxic microenvironment

The hypoxic microenvironment is an important component of the TME and is closely associated with the malignant progression and poor prognosis of CRC (Rainho et al. 2021). The hypoxic microenvironment directly induces proliferation, invasion, metabolism, and genetic instability in CRC (Ulivi et al. 2016). In addition, hypoxia plays a crucial role in driving tumor angiogenesis, and leading to a vicious cycle of hypoxia and angiogenesis within tumors (Ulivi et al. 2016). Previous studies have demonstrated that changes in post-transcriptional m^6^A modifications significantly influence the hypoxic response (Xu et al. 2020a). Recent evidence also suggests that the hypoxic microenvironment acts as novel epigenetic mechanism that promotes CRC metastasis and is closely associated with m^6^A regulators.

**Fig. 3:**
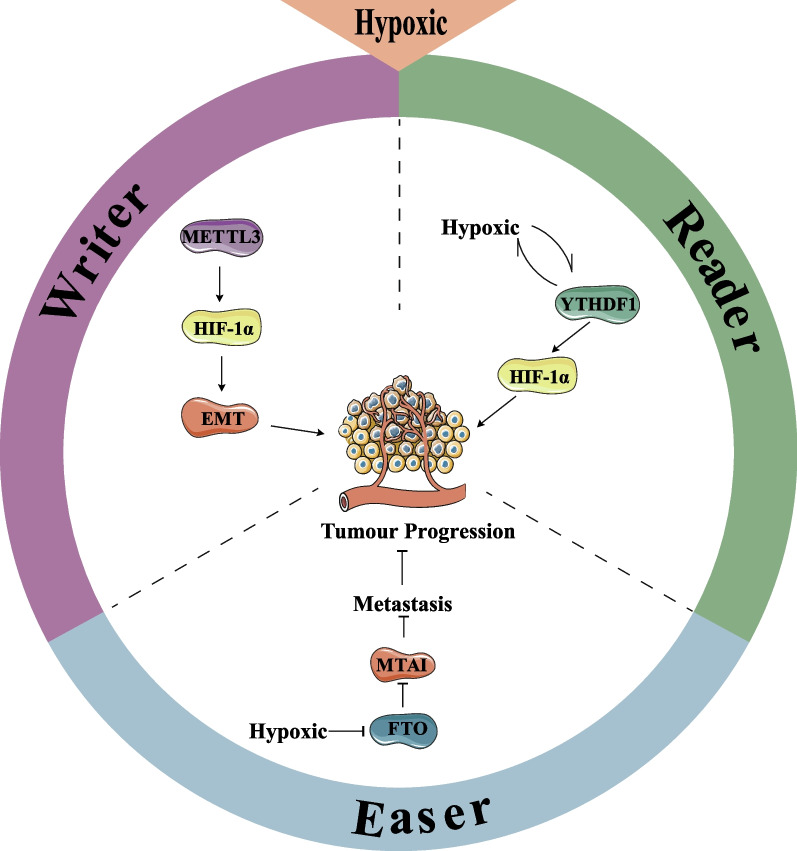
m^6^A modification interacts with the hypoxic microenvironment of CRC. METTL3 mediates the progression of EMT by enhancing the expression of HIF-1α. YTHDF1 is induced by the hypoxic microenvironment and promotes CRC progression by targeting HIF-1α. FTO is inhibited by the hypoxic microenvironment and suppresses CRC metastasis by suppressing the expression of MTA1

Ruan et al*.* found that the m^6^A demethylase FTO was downregulated in CRC, and elevated levels of FTO were associated with a better prognosis for CRC patients. FTO downregulated the transcription of m^6^A downstream target gene MTA1, which inhibits CRC cell growth and metastasis in vivo. However, the context of hypoxic microenvironment decreased the FTO expression and weakened its regulatory activity, which was found to be HIF-1α independent (Ruan et al. 2021). The specific mechanism by which the hypoxic microenvironment downregulates FTO expression remains unclear. Yang et al*.* reported a significant co-expression of METTL3 and HIF-1α, which may be due to METTL3 regulating the translation efficiency of HIF-1α. The expression of METTL3 and total levels of m^6^A were significantly increased in CRC cell lines under hypoxic conditions. METTL3 knockdown inhibited CRC progression under hypoxic conditions. In addition, the m^6^A recognition factor YTHDF1 plays a crucial role as an m^6^A reader by binding to the m^6^A motif, regulating mRNA translation. YTHDF1 was significantly enriched in HIF-1α mRNA under hypoxic microenvironment. The increased expression of YTHDF1 promotes the formation of the hypoxic microenvironment by enhancing HIF-1α expression (Yang et al. 2021). Based on current studies, the hypoxic microenvironment interacts with the biological activity of CRC by regulating m^6^A modifications, and intervention of m^6^A regulators can in turn affect the progression of CRC by regulating the hypoxic microenvironment. Given the close relationship between the hypoxic microenvironment and CRC proliferation and metastasis, m^6^A regulators may be promising predictors and therapeutic targets for CRC prognosis. Figure 3 summarizes the role of m^6^A regulators in the CRC hypoxic microenvironment.

### Inflammatory microenvironment

Chronic inflammation plays a key role in tumor progression, as demonstrated by various epidemiological and experimental studies. The inflammatory microenvironment has been closely associated with the development of CRC. Systemic inflammation is a poor prognosis marker in approximately 20–40% of CRC patients (Park et al. 2017). Inflammatory bowel disease (IBD) is also an independent risk factor for CRC, with inflammatory cells, cytokines and their associated inflammatory signaling pathways contributing to the establishment of an intestinal inflammatory microenvironment (Zhang and Qiao 2022). Increased m^6^A methylation has been demonstrated in major inflammatory pathways, including IL-6, TNF, and NF-κB signaling pathways. Additionally, m^6^A regulators are also involved in regulating inflammatory response of the tumor cells (Hou et al. 2019), (Chokkalla et al. 2019).

Bioinformatic analyses have revealed extensive interactions between m^6^A regulators and IBD risk genes. Moreover, m^6^A-related genes are significantly altered in IBD, and the IBD risk locus is also modified by m^6^A (Nie et al. 2021; Sebastian-delaCruz et al. 2020). Those findings suggested that m^6^A modifications regulated intestinal inflammatory environment are closely associated with the development of IBD. The persistent inflammatory microenvironment may increase the expression of m^6^A and m^6^A regulatory proteins. Cluster analysis based on m^6^A features revealed that subgroups of m^6^A_reg_C1 (characterized with high expression of IGF2BP3) and m^6^A_sig_C1 (characterized with immune activation, with high CD8^+^ effector T cells, transcripts of immune activation, and immune checkpoints) were characterized by activation of inflammatory pathways and infiltration of inflammatory cells, and these subgroups were more responsive for CRC immunotherapy (Zhang et al. 2022b). Therefore, assessing m^6^A levels in CRC could provide insights into the inflammatory microenvironment and help in modulating it for better immunotherapy outcomes. Furthermore, the m^6^A methyltransferase METTL3 has been found to promote the formation of an inflammatory microenvironment and CRC cell proliferation by inhibiting SOCS2 (Li et al. 2021b; Xu et al. 2020b). Additionally, m^6^A methylation of EphA2 and VEGFA regulated by IGF2BP2/3, induces an inflammatory microenvironment and promotes angiogenesis in CRC via PI3K/AKT and ERK1/2 signaling pathway (Liu et al. 2022a). Macrophages play a crucial role in the inflammatory response, with two polarized subtypes, M1 and M2. M1 macrophages exhibit pro-inflammatory and anti-tumor phenotypes, while M2 macrophages exert anti-inflammatory effects and are involved in tumor metastasis (Li et al. 2018). Elevated activity of METTL3 has been found to directly methylate and stabilize STAT1 mRNA, leading to M1 macrophage polarization and inhibition of M2 macrophage formation (Liu et al. 2019).

**Fig. 4:**
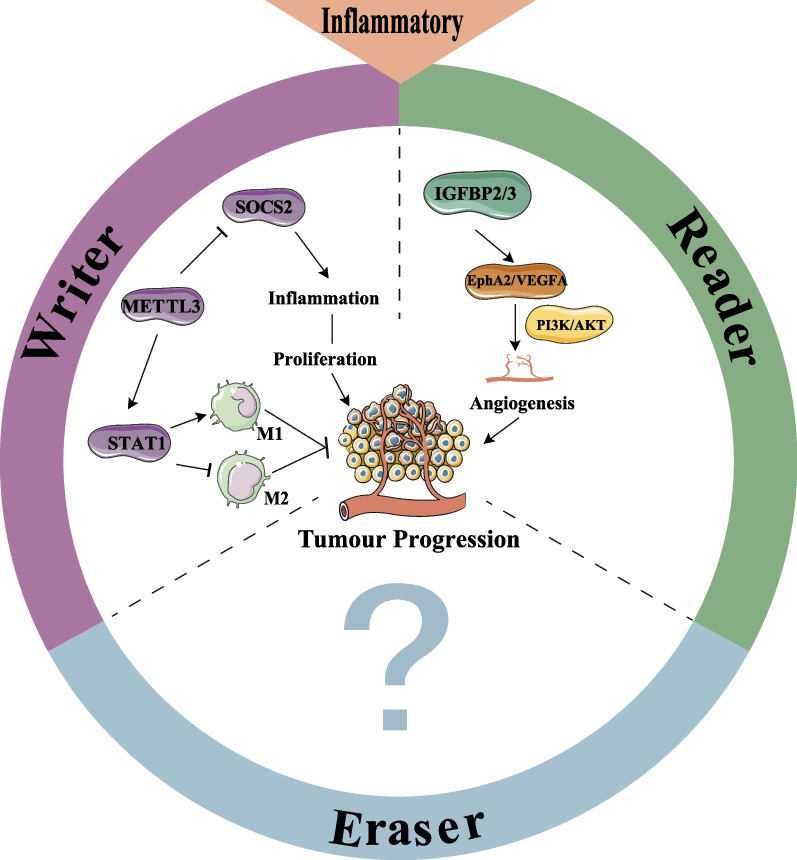
m^6^A modifications interact with the CRC inflammatory microenvironment. METTL3 promotes the formation of the inflammatory microenvironment and CRC proliferation by inhibiting SOCS2. Additionally, METTL3 promotes M1-type macrophage polarization and inhibits M2-type macrophage by promoting STAT1 expression. The m^6^A methylated EphA2/VEGFA promotes angiogenesis in CRC by targeting the PI3K/AKT inflammatory signaling pathway. The functions of m^6^A “erasers” in the inflammatory microenvironment of CRC are still unclear

Together, m^6^A modification regulates the formation of the inflammatory microenvironment, while the inflammatory microenvironment also influences m^6^A modification. However, the most current studies on m^6^A modifications and the inflammatory microenvironment of CRC are based on bioinformatics analysis and require further validation in vitro and in vivo studies. Figure 4 provides and overview of the role of m^6^A regulators in the CRC inflammatory microenvironment.

### Immune microenvironment

TME alters the proliferation, metastasis, and prognosis of CRC. For instance, it induces immune tolerance and immunosuppression, enabling CRC cells to evade the immune system (Shen et al. 2020). The immune components of TME present attractive targets for cancer therapy across various types of cancer. Emerging studies indicated that m^6^A methylation plays a crucial role in regulating the immune microenvironment in CRC.

T cells play a crucial role in regulating the entire adaptive immune response. Recent studies have shown that m^6^A can influence the selective differentiation of tumor-infiltrating T cells by targeting various protein components or signaling pathways (Li et al. 2021c). For instance, increased m^6^A methylation mediated by xeroderma pigmentosum complementation group G (XPG) leads to the release of IFN-γ from Th1 cells. This in turn induces CTL activity and activates CD8^+^CTL (Pal et al. 2022). Additionally, m^6^A targets the SOCS protein family, regulates the IL-7 and TCR signaling pathways, and influences the direction of homeostatic proliferation and differentiation of naive T cells (Li et al. 2017b). Dong et al*.* found that decreased levels of overall m^6^A and METTL14 in CRC tumor stromal cells were associated with reduced T cell infiltration in CRC patients. Interestingly, this study also revealed that repressive macrophages from CRC patients interacted with CD8^+^ T cells in TME (Dong et al. 2021). The immunosuppressive TME inhibits the cytotoxic T cells function and promotes T cell exhaustion, ultimately leading to tumor evasion. Another clinical study has demonstrated that METTL14 deficiency is associated with a poor prognosis in CRC patients (Yang et al. 2020). LncRNA XIST, a target of METTL14, is closely associated with T-cell immunity. XIST regulates the immune function of CD8^+^T cells through the miR-34a-5p/PDL1 axis and promotes Th17 differentiation through the KDM6A-TSAd pathway (Li et al. 2022b; Syrett et al. 2019). Deletion of METTL14 significantly reduces the m^6^A level of XIST, leading to increased XIST expression and enhanced proliferative and invasive abilities of CRC cells (Yang et al. 2020). However, conflicting results have also been reported. Wang et al*.* found that deficiency of METTL3/14 in CRC cells stabilizes SATA1 through YTHDF2. This stabilization leads to increased IFN-γ secretion and CD8^+^T cell infiltration (Wang et al. 2020b). Myeloid-derived suppressor cells (MDSCs) are known for their strong immunosuppressive activities, and promotes the formation of immunosuppressive microenvironment. Chen et al*.* found that METTL3 promotes the expression of BHLHE41 in an m^6^A-dependent manner, which induces CXCL1 transcription and enhances the migration of MDSCs in vitro. Inhibition of METTL3 expression in CRC cells reduces the MDSCs accumulation, maintains the activation and proliferation of CD4^+^ T cells and CD8^+^ T cells, and inhibits the progression of CRC (Chen et al. 2022). Therefore, targeting MDSCs by m^6^A methylation modifications could be a promising strategy for anti-cancer therapy. The inconsistencies observed in these studies may be caused by differences in the downstream targets and modification sites of METTL3 and METTL14. However, the alteration of m^6^A affects the immune microenvironment of CRC, confirming the critical role of m^6^A "readers" in tumor immune surveillance. DCs are responsible for antigen processing, presentation and activation of the T-cell immune response. Meanwhile, a large number of aberrant m^6^A modifications have been found in DCs of tumors (Shulman and Stern-Ginossar 2020). Deletion of YTHDF1 in DCs enhances cross-presentation of CRC antigens and activates CD8^+^T cells in vivo. YTHDF1-deficient CRC mice also exhibit higher sensitivity to immunotherapy (Han et al. 2019a). In addition, CD34 and CD276 have been reported as molecular predictors for the viability of CRC patients, reshaping the immune microenvironment of CRC in an m^6^A-dependent manner, and mediating the immune escape mechanism of CRC by regulating immune checkpoints such as CTLA-4 (Zhou et al. 2021).

**Fig. 5:**
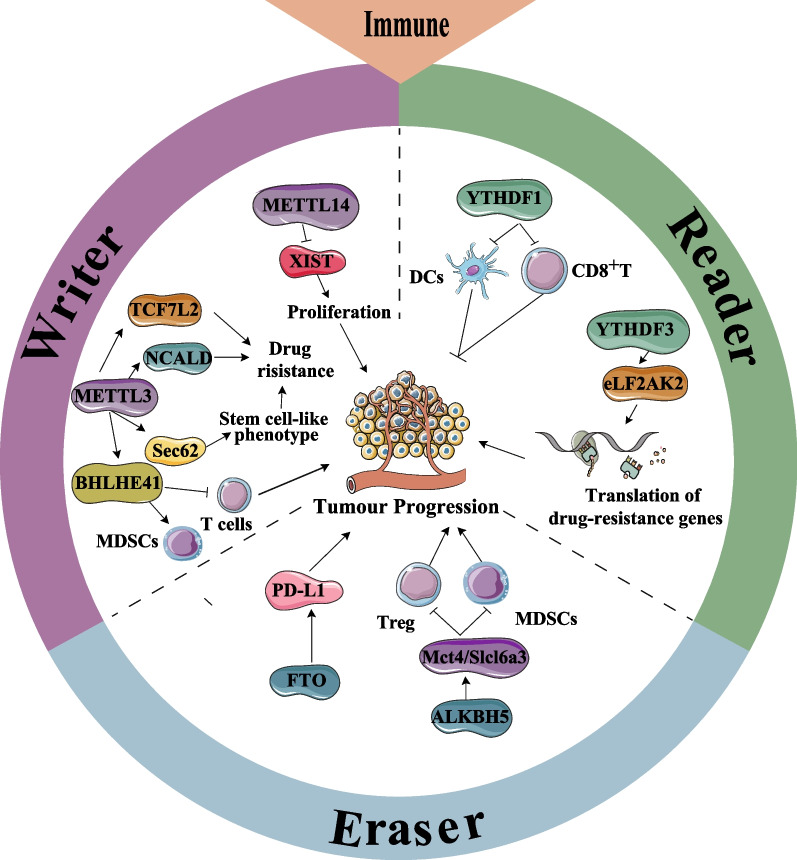
m^6^A modifications interact with the immune microenvironment of CRC. In m^6^A “writers”, METTL3 promotes infiltration of MDSCs and suppresses CD8^+^ T cells by targeting BHLHE41. METTL3 promotes the stem cell-like phenotype of CRC by targeting Sec62. METTL3 promotes CRC progression by targeting NCALD and TCF7L2. METTL14 represses the proliferation of CRC by targeting XIST expression. In m^6^A “Readers”, YTHDF1 inhibits DCs antigen presentation and CD8^+^T cells activation. YTHDF3 promotes the translation of drug-resistance genes by recruiting eLF2AK2. In m^6^A “Erasers”, inhibition of FTO deregulates PD-L1 expression and CRC progression. ALKBH5 inhibits the infiltration of Tregs and MDSCs and the progression of CRC by targeting Mct4/Slc16a3

Together, m^6^A regulators play crucial roles in the formation of the diversity and complexity of the immune microenvironment, and regulate immunosuppression and/or immune escape. To further unravel the relationship between m^6^A modification and immune microenvironment, it is necessary to investigate the effects of m^6^A methylation on the functional and biological behavior of immune cells (e.g., metabolism), as well as the mechanisms of cross-talk between tumor cells, immune cells, and additional stromal cells. The role of m^6^A including the m^6^A-associated immune regulatory network, also requires further investigation in the immune microenvironment of CRC. Figure 5 illustrates the role of m^6^A regulators in the immune microenvironment of CRC.

## Therapeutic strategies for CRC based on m^6^A modification

### Immunotherapy

Immunotherapy is a promising cancer treatment that assists the immune system against tumor cells. The current immunotherapy strategies include monoclonal antibodies, lysing viruses, tumor vaccines, immune checkpoint inhibitors (ICIs), and adoptive transfer. Particularly, immune checkpoint inhibitors have shown remarkable effects. Additionally, the m^6^A modification involved in ICI immunotherapy provides alternative approaches for CRC treatment (Shriwas et al. 2020), (Lichtenstern et al. 2021).

Inhibition of METTL3/14 improves the sensitivity of CRC to anti-PD-1 treatment by impairing m^6^A modification, thereby altering the TME and CD8^+^ T cells recruitment (Wang et al. 2020b). Li et al*.* discovered that m^6^A demethylase ALKBH5-deficient CRC mice exhibited significantly elevated viability after anti-PD-1 treatment. Although ALKBH5 is not required for CRC growth and survival in vivo or in vitro, it plays a critical role in the effectiveness of anti-PD-1 therapy (Li et al. 2020b). The expression of m^6^A recognition protein YTHDF1 correlates with the outcome of immunotherapy in CRC patients. YTHDF1 deletion strengthens the anti-tumor effect of PD-1 blockers by restoring the infiltration of CD8^+^T cells (Li et al. 2023b). The m^6^A demethylase FTO is associated with the progression of various tumors. FTO deletion inhibits PD-L1 expression in CRC cells, and this process is independent of IFN-γ signaling (Tsuruta et al. 2020). Furthermore, numerous bioinformatics analyses have revealed that m^6^A modifications are involved in shaping tumor immune microenvironment profiles (Chen et al. 2021a), (Liu et al. 2022b). All the findings highlight the significant role of m^6^A modifications in modulating the responsiveness of CRC to immunotherapy, and the m^6^A modulators may serve as potential therapeutic targets for CRC alone or in combination with immune checkpoint inhibitors.

### Chemotherapy

Drug resistance is the primary cause of failure in cancer chemotherapy. The mechanisms underlying tumor drug resistance include in drug metabolism, tumor heterogeneity, microenvironmental alterations, and mutations in target proteins (Vasan et al. 2019).

Oxaliplatin (OX) is widely used first-line chemotherapeutic agent for cancer treatment, but resistance developed by tumor cells poses a major challenge in the treatment of advanced CRC. Lan et al*.* discovered that the total m^6^A content and the expression of methyltransferase METTL3 increased in CRC tissues from OX-resistant patients. TAM in TME contribute to OX-resistance in CRC cells via METTL3-mediated m^6^A modification (Lan et al. 2021). METTL3-induced m^6^A modification increases the stability of Sec62 mRNA and upregulates Sec62 expression, which maintains the stem cell-like phenotype and chemotherapy resistance in CRC (Liu et al. 2021). IR100HG stabilizes TCF7L2 mRNA with METTL3-mediated m^6^A modification to regulate CRC resistance to cetuximab. TCF7L2 in turn regulates the transcription of MIR100HG and blocks the positive feedback pathway between them (Gao et al. 2021; Liu et al. 2022c). METTL3 is associated with 5-fluorouracil (5-FU) resistance in CRC. METTL3-mediated m^6^A modification acts on DiGeorge syndrome critical region 8 (DGCR8) in CAFs, promoting the secretion of exosomal miR-181b-5p, which inhibits CRC sensitivity to 5-FU by targeting NCALD (Pan et al. 2022). Moreover, the YTHDF family has also been implicated in drug resistance in CRC. Expression of YTHDF1 is significantly upregulated in cisplatin-resistant CRC cell lines and promotes CRC resistance to cisplatin by mediating glutamine metabolism (Chen et al. 2021b). Moreover, YTHDF1 is targeted by miR-136-5p to mediate CRC chemoresistance (Jiang et al. 2022). YTHDF3 is highly expressed in OX-resistant CRC tissues and recognizes the 5′-UTR of m^6^A-methylated RNAs associated with tumor resistance, and recruiting eukaryotic translation initiation factor 3 subunit A (eIF3A) to promote translation of drug-resistant genes (Zhao et al. 2022). All the studies demonstrate that m^6^A modification mediates chemoresistance in CRC by altering various cell types in the TME. Consequently, targeting m^6^A regulators may offer a new horizon for addressing chemoresistance of CRC.

## Discussion

TME plays a critical role in the development of human cancers (Hinshaw and Shevde 2019). The prevalence and aberrant distribution of m^6^A modifications are involved in tumor development (Li et al. 2021d; Han et al. 2019b). The “writers”, “erasers”, and “readers” of m^6^A dynamically regulate the components of the TME through complex pathways. These pathways influence the metabolic, hypoxic, inflammatory, and immune microenvironment (Fang et al. 2022). As m^6^A regulators affect multiple tumorous microenvironments, blocking one pathway may result in compensatory expression in another pathway. This highlights the need for co-blockade of multiple pathways to achieve synergistic anti-tumor activity. Table 1 provides a summary of the effects and targets of m^6^A regulators in the CRC microenvironment.

**Table 1:** Functions of m6A related enzymes and targets in CRC

Molecules	Type	Related Microenvironment	Targets	Effects	References
METTL3	Writer	Metabolic	CCNE1	Interregulate with intestinal metabolites and promote CRC proliferation	Zhu et al. (2020)
METTL3	Writer	Metabolic	PTTG3P	Induce glycolysis and CRC proliferation	Zheng et al. (2021)
METTL3	Writer	Metabolic	LDHA	Regulate glucose metabolism in CRC, and mediate the resistance of CRC cells to 5-FU	Zhang et al. (2022a)
METTL14	Writer	Metabolic	miR-149-3p	Promote intestinal inflammation and CRC	Cao et al. (2021b)
KIAA1429	Writer	Metabolic	HK2	Accelerate aerobic glycolysis and the production of malignant phenotypes in CRC	Li et al. (2022a)
YTHDF1	Reader	Metabolic	GLS	Promote CRC resistance to cisplatin	Chen et al. (2021b)
YTHDF2	Reader	Metabolic	DEGS2	Inhibit dysregulation of lipid metabolism and proliferation and migration of CRC	Guo et al. (2021)
IMP2	Reader	Metabolic	ZFAS1	Mediate the energy metabolism of cell mitochondria, promote the proliferation of CRC and inhibit the apoptosis of CRC cells	Lu et al. (2021)
METTL3	Writer	Hypoxic	HIF-1a	Promote the progression of CRC under hypoxia	Yang et al. (2021)
YTHDF1	Reader	Hypoxic	HIF-1a	Promote the formation of the hypoxic microenvironment and the progression of CRC	Yang et al. (2021)
FTO	Eraser	Hypoxic	MTA1	Inhibit CRC cell growth and metastasis in vivo	Ruan et al. (2021)
METTL3	Writer	Inflammatory	SOCS2	Induce CRC cell proliferation and maintain tumorigenicity of CRC	Xu et al. (2020b)
METTL3	Writer	Inflammatory	STAT1	Promote M1-type macrophage polarization and inhibit M2-type macrophage formation	Liu et al. (2019)
IGF2BP2/3	Reader	Inflammatory	EphA2, VEGFA	Promote angiogenesis in CRC	Liu et al. (2022a)
METTL3	Writer	Immune	Sec62	Maintain the stem cell-like phenotype and resistance to chemotherapeutic agents in CRC	Liu et al. (2021)
METTL3	Writer	Immune	TCF7L2	Regulate CRC resistance to cetuximab	Liu et al. (2022c)
METTL3	Writer	Immune	NCALD	Inhibit CRC sensitivity to 5-FU	Pan et al. (2022)
METTL3	Writer	Immune	BHLHE41	Enhance the migration of MDSCs in vitro and inhibit the activation and proliferation of CD4^+^ T and CD8^+^ T cells	Chen et al. (2022)
METTL14	Writer	Immune	–	Regulate infiltration of CD8^+^T cells	Dong et al. (2021)
METTL14	Writer	Immune	lncRNA XIST	The deletion of this gene can promote the proliferation and invasion of CRC	Yang et al. (2020)
METTL3/METTL14	Writer	Immune	STAT1	The deletion of these genes can promote IFN-γ secretion and increase CD8^+^T cell infiltration, thus improving the sensitivity of CRC against PD-1 treatment	Wang et al. (2020b)
YTHDF1	Reader	Immune	–	The deletion of this gene can improve the antigen presentation ability of DCs and activation of CD8^+^ T cells in vivo	Shulman and Stern-Ginossar (2020)
YTHDF3	Reader	Immune	eLF2AK2	Promote translation of drug-resistant genes	Zhao et al. (2022)
ALKBH5	Eraser	Immune	Mct4/Slc16a3	Regulate infiltration of Tregs and MDSCs, plays a critical role in the efficacy of anti-PD-1 therapy	Li et al. (2020b)
FTO	Eraser	Immune	–	Gene deletion inhibits PD-L1 expression in CRC cells	Tsuruta et al. (2020)

In addition, m^6^A modifications enable cross-talk with different microenvironments. Metabolites produced by gut microbes regulate the levels of m^6^A in different cell types, consequently affecting cellular activity in TME. Furthermore, alterations in metabolism, induced by the metabolic microenvironment and hypoxic condition, contribute to the development of a chronic inflammatory microenvironment. This, in turn, suppresses immune function within the gut and provides favorable conditions for progression of CRC. The m^6^A regulatory factor exhibits diverse effects in different microenvironments, and its response functions can be considered a double-edged sword in CRC. For instance, METTL3 has been proposed as a tumor-promoting factor, whereas it also plays a role in polarizing M1 macrophages and exhibits anti-tumor effects (Gunassekaran et al. 2021).

Exosomes facilitate the transfer and exchange of miRNAs, mRNAs, and lncRNAs between cells and tissues, and have been suggested to play a critical role in regulating the TME (Wortzel et al. 2019). Interestingly, recent studies have proposed that the inter-regulatory relationship between exosomes and m^6^A is associated with tumor tolerance to chemotherapy and radiotherapy, possibly by influencing the TME (Song et al. 2021). Additionally, the key to successful therapy targeting the m^6^A enzyme lies in safely and efficiently delivering the therapeutic agent to specific cells. Firstly, these carriers must protect the cargo from destruction, and secondly, they must bind to specific cells and enter them to release the cargo. This requires an effective drug delivery route and specific targeting molecules on the carrier surface to attract receptors on the target cell surface. Taking these conditions into account, viral delivery systems, lipid nanoparticle delivery systems, and virus-like particle delivery systems show promise for targeting m^6^A enzymes in specific cells (Raguram et al. 2022). Currently available techniques for measuring m^6^A activity include high-throughput sequencing, colorimetry, and liquid chromatography-mass spectrometry, such as MeRIP-seq, miCLIP-seq, SCARLET, and LC–MS/MS. Using these techniques to measure the m^6^A activity of specific genes in CRC cells holds potential as a diagnostic tool for monitoring the progress of CRC.

Although the emergence of m^6^A regulators has provided new ideas for CRC treatment, there are still numerous challenges regarding the regulatory role of m^6^A modifications on TME and related applications. Firstly, m^6^A modifications are abundant in TME, but most studies have not detected specific biological functions of the associated regulators, limiting further exploration of their applications. Secondly, since RNA status varies across individuals, tissues, and cell types, it is difficult to target m^6^A modifications to specific cell types in different individuals. Additionally, m^6^A modification is a complex regulatory network, and the current understanding of the relevant regulatory factors of m^6^A is incomplete, leaving more m^6^A “writers”, “erasers” and “readers” to be uncovered. Finally, few TME-related studies in CRC currently involve the process of m^6^A modification in vivo, with most being bioinformatics analyses or in vitro assays. In contrast, m^6^A modification is a dynamic regulatory process in vivo, posing a major obstacle to applying relevant findings to clinical practice.

## Conclusion

The TME is a complex and dynamic system that encompasses hundreds of chemical modifications. These modifications serve to activate and influence signaling mechanisms for epigenetic changes. Among these modifications, the m^6^A modifications play a crucial role in regulating the dynamics of the TME and have a profound impact on the metabolic, hypoxic, inflammatory, and immune microenvironment of CRC. The m^6^A modulators have extensive applications in settings and show great potential as novel biomarkers or targets for interventions in CRC. Therefore, it is essential to further comprehend the regulatory mechanisms of m^6^A modifications in the TME in order to explore the oncogenes and biological behaviors associated with CRC.

## Data Availability

Not applicable.

## Abbreviations

5-FU: 5-Fluorouracil
ALKBH3: α-Ketoglutarate-dependent dioxygenase homolog 3
ALKBH5: α-Ketoglutarate-dependent dioxygenase homolog 5
CAFs: Cancer-associated fibroblasts
CBLL1: Cbl proto-oncogene, E3 ubiquitin protein ligase-like 1
CCNE1: Cell cycle protein E1
CRC: Colorectal cancer
DCs: Dendritic cells
DGCR8: DiGeorge syndrome critical region 8
eLF2AK2: Eukaryotic translation initiation factor 3 subunit A
ETBF: Enterotoxigenic Bacteroides Fragilis
FTO: Fat mass and obesity-associated protein
HIF-1α: Hypoxia-inducible factor 1-α
HNRNPA2B1: Heterogeneous nuclear ribonucleoprotein A2B1
HNRNPC: Heterogeneous nuclear ribonucleoprotein C
IBD: Inflammatory bowel disease
IGF2BPs: Insulin-like growth factor 2 mRNA-binding proteins
IMPs: IGF-II mRNA-binding proteins
m^6^A: N^6^-methyladenosine
MDSCs: Myeloid-derived suppressor cells
METTL14: Methyltransferase-like 14
METTL16: Methyltransferase-like 16
METTL3: Methyltransferase-like 3
ncRNAs: Non-coding RNAs
OX: Oxaliplatin
RBM15: RNA-binding motif protein 15
RBM15B: RNA-binding motif protein 15B
TAMs: Tumor-associated macrophages
TANs: Tumor-associated neutrophils
TME: Tumor microenvironment
UC: Ulcerative colitis
WTAP: Wilms’ tumor 1-associated protein
YTHDC: YT521-B homology domain containing proteins
YTHDF: YT521-B homology domain family proteins
ZC3H13: Zinc finger CCCH-type containing 13
